# Recent Advances in Solid Tumor CAR-T Cell Therapy: Driving Tumor Cells From Hero to Zero?

**DOI:** 10.3389/fimmu.2022.795164

**Published:** 2022-05-11

**Authors:** Pouya Safarzadeh Kozani, Pooria Safarzadeh Kozani, Milad Ahmadi Najafabadi, Fatemeh Yousefi, Seyed Mohamad Javad Mirarefin, Fatemeh Rahbarizadeh

**Affiliations:** ^1^ Department of Medical Biotechnology, Faculty of Paramedicine, Guilan University of Medical Sciences, Rasht, Iran; ^2^ Department of Medical Biotechnology, Faculty of Medical Sciences, Tarbiat Modares University, Tehran, Iran; ^3^ Department of Genetics, Faculty of Biological Sciences, Tarbiat Modares University, Tehran, Iran; ^4^ Department of Immunology, Faculty of Medical Sciences, Tarbiat Modares University, Tehran, Iran; ^5^ Research and Development Center of Biotechnology, Tarbiat Modares University, Tehran, Iran

**Keywords:** chimeric antigen receptor, immunotherapy, solid tumors, infiltration, vaccines, tumor microenvironment

## Abstract

Chimeric antigen receptor T-cells (CAR-Ts) are known as revolutionary living drugs that have turned the tables of conventional cancer treatments in certain hematologic malignancies such as B-cell acute lymphoblastic leukemia (B-ALL) and diffuse large B-cell lymphoma (DLBCL) by achieving US Food and Drug Administration (FDA) approval based on their successful clinical outcomes. However, this type of therapy has not seen the light of victory in the fight against solid tumors because of various restricting caveats including heterogeneous tumor antigen expression and the immunosuppressive tumor microenvironments (TME) that negatively affect the tumor-site accessibility, infiltration, stimulation, activation, and persistence of CAR-Ts. In this review, we explore strategic twists including boosting vaccines and designing implementations that can support CAR-T expansion, proliferation, and tumoricidal capacity. We also step further by underscoring novel strategies for triggering endogenous antitumor responses and overcoming the limitation of poor CAR-T tumor-tissue infiltration and the lack of definitive tumor-specific antigens. Ultimately, we highlight how these approaches can address the mentioned arduous hurdles.

## 1 Introduction

Immune checkpoints are known as naturally occurring pathways that prevent the immune system from attacking and destroying healthy and domestic cells in the body (1). Cancer cells leverage this immune action mechanism to escape from the harm of the immune system (1). If cancer cells do not use these immune checkpoints as a protection shield, the immune system might attack and destroy them (1). Immune checkpoint inhibitors are a type of solid tumor cancer therapy (1–4). These agents put the brakes on the immune checkpoints and unleash immune responses against tumors (including T-cell responses) (1–4). The mentioned reactions following immune checkpoint blockade therapy can result in effective tumor eradication, as demonstrated in various studies (1–4). However, there are some limitations regarding this type of therapy as it has been evident that it cannot result in an adequate number of tumor-reactive T cells (1, 5). Moreover, the triggered antitumor responses are often weak and memory T cell formation is not effectively carried out (1, 5).

Adoptive cellular therapy (ACT) is another field of cancer immunotherapy that includes obtaining a patient’s or donor’s cells, modifying or expanding them in *ex vivo* conditions, and delivering the modified or expanded cells to the patient (6). ACT is mainly categorized into three distinct fields which include tumor-infiltrating lymphocytes (TILs), genetically engineered T-cell receptors (TCRs), and chimeric antigen receptor T cells (CAR-Ts) (7–9). ACT has proven efficient in comparison with immune checkpoint blockade therapy, especially in terms of antitumor T-cell populations and specific responses (7–9). Today’s modern CAR-T therapy is based on the experience gained from early ACT. For instance, in the context of TIL therapy, it was discovered that tumor-site T-cell infiltration is observed in various types of solid tumors (including melanoma, head and neck squamous cell carcinoma, cervical cancer, and lung cancer) and this T-cell infiltration has a direct relationship with patients’ favorable prognosis (10–13). However, it was elucidated that the antitumor activity of these T cells is significantly hampered by the immunosuppressive tumor microenvironment (TME) (14). Later, isolation, expansion, and adoptive transfer of these infiltrating cells was proposed as a therapeutic solution. This approach proved effective in eradicating various types of tumors both in preclinical and clinical studies (7, 11, 15). Moreover, studies demonstrated that both CD4^+^ T cells and CD8^+^ T cells have critical roles in such effective antitumor responses (10, 16–19). In detail, CD8^+^ T cells are able to produce proinflammatory cytokines once they are activated (10, 16–19). Such proinflammatory cytokines can mediate irreversible tumor cell damage (10, 16–19). Moreover, CD4^+^ T cells can help B cells produce antibodies once they develop into antibody-producing plasma cells (10, 16–19). In addition, these cells help trigger CD8^+^ T cell immune responses (10, 16–19).

Moreover, it was also discovered that using interleukin (IL)-2 (its systematic administration or its use for the expansion of TILs) can enhance the antitumor efficacy of *ex vivo*-expanded TILs (15, 20, 21). Such findings implied that the adoptive transfer of *ex vivo*-expanded cells can be both therapeutically safe and beneficial in eliminating solid tumor cells (15, 20, 21). Later, it was revealed that using lymphodepletion chemotherapy alongside ACT can enhance *in vivo* T-cell proliferation and persistence in patients with solid tumors or hematologic malignancies (22, 23). Lymphodepletion chemotherapy is the use of certain chemotherapy agents before the adoptive transfer of *ex vivo*-expanded or modified cells to the recipient. This approach prepares recipients’ immune systems for ACT (22, 23). Various studies have demonstrated that this approach results in improved clinical outcomes of ACT (22, 23).

**Figure 1:**
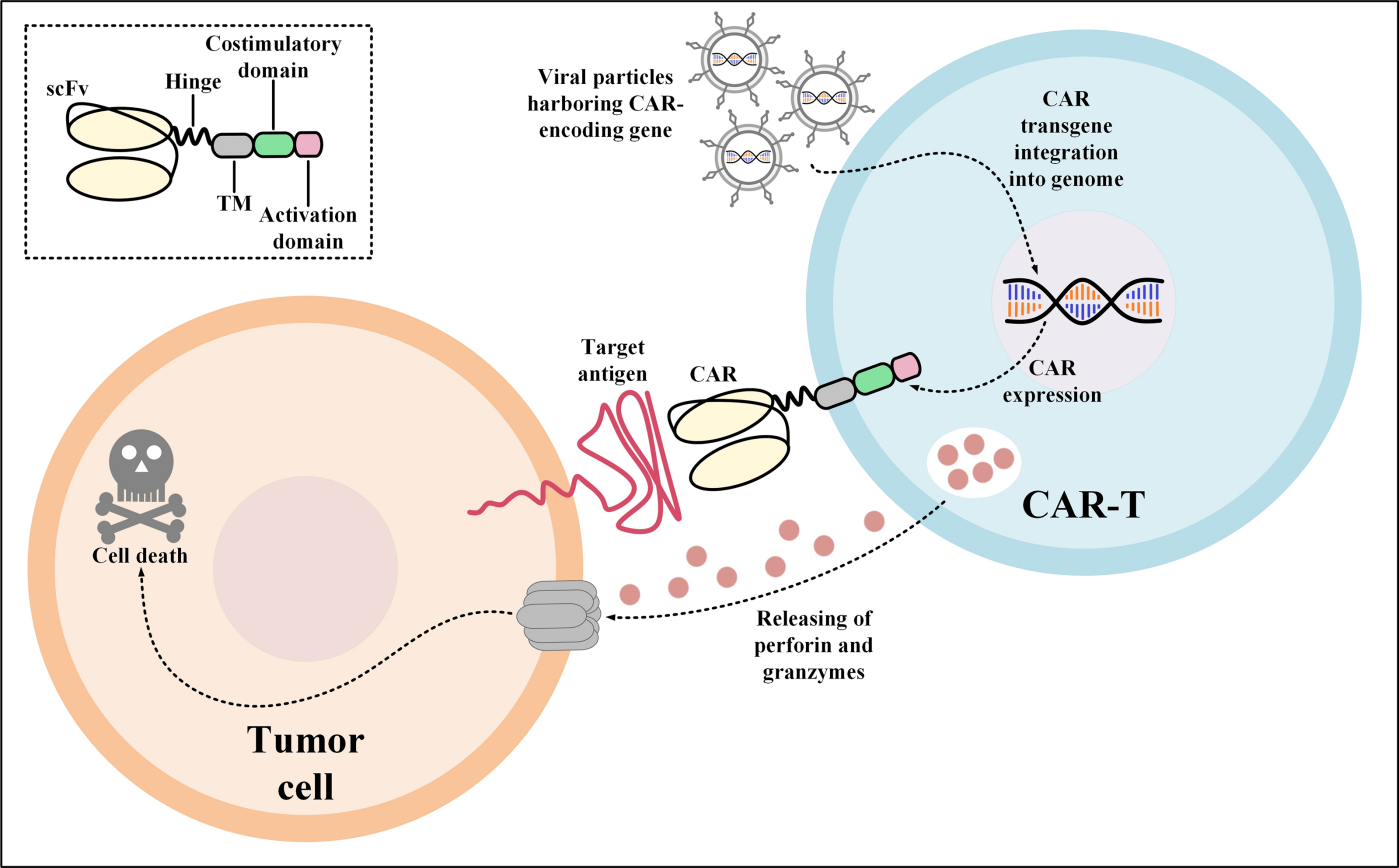
Action mechanism of CAR-Ts for the elimination of target tumor cells expressing the CAR target antigen. Transduction of T cells with viral particles harboring the CAR-encoding transgene leads to the stable expression of CARs on the surface of the transduced T cells. Upon target antigen encountering, CAR-Ts are activated and they release perforin and granzymes leading to tumor cell death. CAR, chimeric antigen receptor; scFv, single-chain variable fragment; TM, transmembrane domain.

CAR-Ts are T cells engineered to express CARs which are versatile synthetic receptors composed of an antigen-targeting domain, mostly derived from the single-chain variable fragment (scFv) of a monoclonal antibody (mAb), fused to an intracellular T-cell activation signaling domain and one or two co-stimulatory domains (24). CARs have the unique ability to redirect engineered T cells towards cancer cells expressing the target antigen of interest (24). CAR-Ts are activated upon interaction with their target antigen which is in a major histocompatibility complex (MHC)-independent manner (**Figure 1**) (24). It has not been a long time since the first CAR-T product (named *tisagenlecleucel*) received US Food and Drug Administration (FDA) approval in 2017 for medical use in clinics (25, 26). Today, CAR-T therapy is available as an efficient treatment option for the treatment of certain patients with relapsed or refractory (R/R) hematologic malignancies including B-cell acute lymphoblastic leukemia (B-ALL), diffuse large B-cell lymphoma (DLBCL), follicular lymphoma (FL), mantle cell lymphoma (MCL), and multiple myeloma (MM) (9, 25–35).

Considering the durable clinical responses in the treatment of patients with the mentioned hematological indications (which still has room for improvement), what came out as bad news was the unfavorable efficacy of CAR-T therapy against solid tumors (36–39). These unsuccessful outcomes are mainly due to the lack of suitable CAR-T target antigens in solid tumors and that the targeted antigens are usually found on healthy tissues leading to *“on-target off-tumor”* toxicities. Other important hindrances include the poor accessibility of the target antigen by CAR-Ts that leads to inefficient CAR-T *in vivo* stimulation, activation, and expansion, the heterogeneous pattern of tumor antigen expression and the appliance of escape mechanisms by tumor cells to evade CAR-Ts redirected towards a single tumor antigen, the immunosuppressive nature of the TME rendering CAR-Ts non-responsive or exhausted, insufficient infiltration of CAR-Ts into the TME, and metabolic starvation (40–42). Ever since the remarkable functionality of CAR-Ts was observed in hematologic malignancies, different types of improvement strategies have been developed to at least bring a fraction of the success of hematologic malignancy CAR-T therapy to the table of solid tumor therapy (43). In this review, we explore the most novel strategies for tackling some of the most important and clinical success-limiting challenges of CAR-T therapy in solid tumors.

## 2 CAR-T Therapy Fundamentals

TCRs expressed on the surface of T lymphocytes are responsible for recognizing the peptide antigens presented to them through MHCs by antigen-presenting cells (APCs). The action mechanism of these receptors only enables mediating immune reactions towards peptide antigens that are presented by MHCs. On the other hand, mAbs can recognize and bind cell surface-expressed antigens that are not presented by MHCs. This ability of mAbs has been utilized for redirecting the cytotoxicity of various kinds of immune cells including T cells and natural killer (NK) cells towards tumor surface-expressed antigens of interest which can be either tumor-specific antigens (TSAs) or tumor-associated antigens (TAAs) (44, 45). CAR molecules are made of three key segments. An extracellular domain (consisting of a targeting domain and a spacer), a transmembrane domain, and an intracellular domain (consisting of one or two costimulatory domains and a primary activation domain) (46, 47).

CAR-T development has experienced a fast-paced journey from its early days that has led to the production of CAR-Ts with versatile capabilities for tackling various CAR-T therapy obstacles (46, 48). In terms of structural designing, CAR-Ts are currently classified into five generations, each with different structural components and rather distinct biological behaviors. The first generation of CAR-Ts expressed extracellular antigen-targeting domains fused to an intracellular domain through a transmembrane domain (49). These CAR-Ts demonstrated insignificant expansion, persistence, and clinical responses in clinical studies (49, 50). Such clinical failures persuaded researchers to develop CAR-Ts capable of overcoming these limitations. In this regard, the second and third generations of CAR-Ts were designed which harbored one and two costimulatory domains in their intracellular domain, respectively (51, 52). These generations of CAR-Ts exhibited significant capabilities in tackling the hurdles of first-generation CAR-Ts and they proved efficient and safe in clinics (9, 27–32, 51, 52). It is worth mentioning that all of the FDA-approved CAR-T products are second-generation CAR-Ts harboring either 4-1BB or CD28 as their costimulatory domains (9, 27–32). Furthermore, researchers used the backbone of second-generation CAR-Ts and modified its intracellular domain by the fusion of additional domains to achieve different goals (53–55). In this regard, the fourth and the fifth generation of CAR-Ts were developed. In detail, the fourth-generation CAR-Ts, alternatively termed as “*T-cell redirected for universal cytokine-mediated killing (TRUCKs)*” or *armored CAR-Ts*, harbor an intracellular expression inducer of a cytokine of interest (which endows them with the capability of targeted delivery of a cytokine of interest) whereas the fifth-generation CAR-Ts have an intracellular fragment of a cytokine receptor (for instance, IL-2Rβ) (53–55). All of these basic CAR designing twists have been carried out on the grounds of achieving superior CAR-T-mediated antitumor responses and enhanced safety profiles. In the upcoming sections, we highlight studies that have specifically aimed to find strategies capable of tackling particular limitations of CAR-T therapy in solid tumors.

## 3 The Hurdles of Solid Tumor CAR-T Therapy

**Figure 2:**
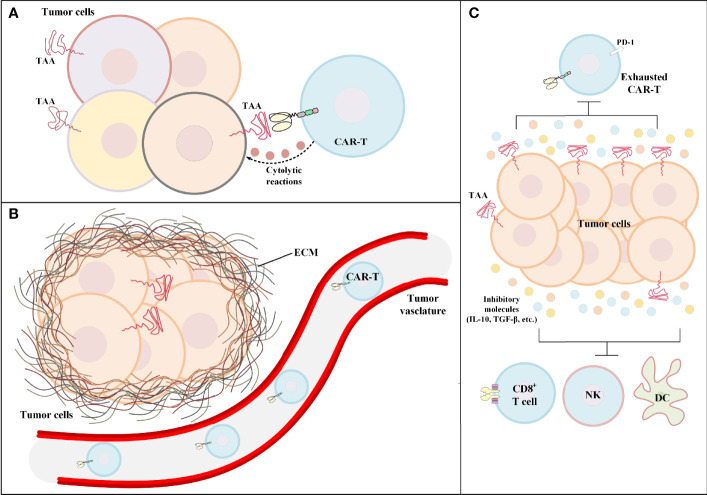
The hurdles of CAR-T therapy in solid tumors. **(A)** Tumor antigen heterogeneity. In such conditions, the tumor bulk consists of tumor cells only a fraction of which express the CAR-redirected TAA on their surface. Other tumor cells may express different TAAs. **(B)** Poor tumor site CAR-T infiltration. In the case of hematologic malignancies, the adoptively transferred CAR-Ts encounter their target cells in the bloodstream or the lymphatic system. On the contrary, in solid tumor CAR-T therapy, CAR-Ts have limited accessibility to the tumor site rendering CAR-T-mediated tumoricidal reactions ineffective and insufficient. **(C)** The immunosuppressive tumor microenvironment. The immunosuppressive characteristics of the tumor microenvironment have negative effects on both CAR-Ts and various immune cells responsible for endogenous antitumor responses. CAR-T, chimeric antigen receptor T cell; DC, dendritic cell; ECM, extracellular matrix; IL-10, interleukin-10; NK, natural killer cell; PD-1, programmed cell death receptor protein 1; TAA, tumor-associated antigen; TGF-β, transforming growth factor-beta.

CAR-T therapy of solid tumors has not been very successful in the clinics. Identifying the hurdles of solid tumor CAR-T therapy is the first and the most important step for developing counterstrategies for tackling these limitations. One of the primary limitations of solid tumor CAR-T therapy is target antigen heterogeneity. CAR target antigen heterogeneity results in poor detection of cancer cells by CAR-Ts and leads to inadequate CAR-T-mediated antitumor reactions against cancer cells expressing that particular type of target antigen (**Figure 2A**). Such ineffective antitumor responses can result in treatment failure and further tumor outgrowth. Moreover, in solid tumor CAR-T therapy, most targeted antigens are not essential for tumor progression; therefore, the expression of such antigens can be reduced by tumor cells in an intelligent manner (56). Various studies have demonstrated that low-level CAR-T target antigen expression is also an important factor for the ineffectiveness of solid tumor CAR-T therapy and the occurrence of disease relapse (36, 57). For instance, one study reported that low-density expression of target antigen resulted in the inability of CAR-Ts for tumor eradication and also the occurrence of disease relapse in preclinical models of pancreatic and prostate cancer (57). Moreover, according to the results of a clinical study, in some solid tumors, CAR-T-mediated targeting of a particular target antigen may result in its downregulation rendering CAR-T therapy incapable of attacking tumor cells, and leading to treatment failure (36). In detail, O’Rourke et al. reported that in the first-in-human pilot study of epidermal growth factor receptor variant III (EGFRvIII)-redirected CAR-Ts in refractory glioblastoma patients, it was discovered that patient-obtained tumor biopsies following CAR-T therapy had reduced the expression level of EGFRvIII in comparison with those biopsies obtained before CAR-T therapy (36).

In the case of hematologic malignancies, CAR-Ts encounter their target cells in the bloodstream and the lymphatic system, and that is where most of the fight against tumors takes place. But in the case of solid tumors, the story is quite different. There is scientific evidence indicating that different tumor-applied mechanisms result in the declined secretion of various vasculature-related factors rendering CAR-Ts unable to pass through the vascular endothelium and penetrate the tumor tissue (**Figure 2B**) (58). For instance, in a study by Vedvyas et al. investigating the preclinical efficacy of intercellular adhesion molecule-1 (ICAM-1)-redirected CAR-Ts for targeting advanced thyroid cancer, it was elucidated that endothelin B receptors are upregulated in cancer tissues which results in the reduced ICAM-1 expression level rendering CAR-Ts incapable of crossing the blood vessels (59). Additionally, CAR-T migration, penetration, and trafficking into tumor tissues are highly dependent on various types of chemokines (60). It has been evident that such chemokines are downregulated or not expressed in tumor tissues (60). This mechanism results in the reduced migration and penetration of T lymphocytes (as well as CAR-Ts) into the tumor sites (60). Moreover, the presence of a dense extracellular matrix is known as another barrier for T-cell migration and infiltration (61). It has been demonstrated that controlled degradation of tumor extracellular matrix can result in increased susceptibility of the tumor to CAR-T therapy (62). Other scientific evidence demonstrates that the WNT/β-catenin signaling pathway is responsible for blocking T-cell infiltration in the tumor site in various types of solid tumors (including metastatic melanoma and colorectal cancer) (63–65). This mechanism can result in immune evasion of cancer and resistance to therapy (63–65). In this regard, a deep understanding of solid tumor mechanisms and developing effective strategies to tackle them is an important factor for having an effective CAR-T therapy in solid tumors. In this article, we have tried to discuss some of such strategies.

The territory of solid tumors, known as the immunosuppressive TME, has various characteristics that hamper antitumor immune reactions (including T-cell and CAR-T activity) (**Figure 2C**). An effective solid tumor therapy is based on overcoming the immunosuppressive nature of the TME. There are various types of immune suppressor cells in the tumor environment, including regulatory T cells (Tregs), myeloid-derived suppressor cells (MDSCs), and tumor-associated macrophages (TAMs) (66–69). Alongside immunosuppression, such cells can be beneficial for tumors cells in terms of supporting tumor progression, angiogenesis, and metastasis (66, 68). In detail, these cells assist in such tumor growth-related activities by producing growth factors, chemokines, and cytokines (such as various types of ILs or TGF-β) (66, 68). Studies have demonstrated that immune checkpoint molecules (CTLA-4 and PD-1) may also play important roles in impairing certain antitumor reactions (1, 70–72). Such understanding of the TME and its impact on the CAR-T-mediated antitumor responses has encouraged researchers to find various effective counterstrategies. In the upcoming sections, we mention some of these counterstrategies (some of which have been investigated in clinical trials as summarized in **Table 1**) which might improve the outcomes of solid tumor CAR-T therapy.

**Table 1:** A summary of the advances in solid tumor CAR-T therapy investigated in clinical trials.

Clinical trial identifier (Phase)	Notes	Participants	Start – completion date	Indication(s)	Target antigen
NCT01953900 (Phase I)	Varicella zoster virus (VZV) vaccination to enhance the activity of GD2-VZV-CAR-Ts	26	April 2014 - October 31, 2034	Osteosarcoma, Neuroblastoma	GD2
NCT03602157 (Phase I)	CCR4^+^ CD30-redirected CAR-Ts	59	December 12, 2018 - September 30, 2041	Various lymphomas	CD30
NCT04153799 (Phase I)	CXCR5-expressing EGFR-redirected CAR-Ts	11	November 1, 2019 - December 2022	Non-small cell lung cancer	EGFR
NCT03929107 (Phase II)	IL-7- and CCL19-expressing CAR-Ts	80	March 28, 2019 - April 30, 2022	B-cell lymphoma	CD19
NCT03682744 (Phase I)	Intraperitoneal Infusions of CEA-redirected CAR-Ts for the treatment of peritoneal carcinomatosis	18	September 13, 2018 - March 2021	Peritoneal carcinomatosis	CEA
NCT03389230 (Phase I)	Intratumoral/intracavitary infusion of HER2-redirected CAR-Ts for the treatment of glioma	42	August 14, 2018 - December 15, 2023	R/R malignant glioma	HER2
NCT04661384 (Phase I)	Intracerebroventricular delivery of IL13Rα2-redirected CAR-Ts for the treatment of leptomeningeal metastases	30	March 5, 2021 - December 15, 2023	Leptomeningeal metastases	IL13Rα2
NCT04951141 (Early Phase I)	Intratumoral delivery of GPC3-redirected CAR-Ts for the treatment of hepatocellular carcinoma	10	January 1, 2019 - December 21, 2023	Hepatocellular carcinoma	GPC3
NCT02414269 (Phase I/II)	Intrapleural administration of mesothelin-redirected for the treatment of pleural cancers	113	May 2015 - April 2023	Malignant pleural cancers	Mesothelin
NCT04077866 (Phase I/II)	Intratumoral/intracerebroventricular administration of B7-H3-redirected CAR-Ts	40	May 1, 2022 - July 1, 2024	R/R glioblastoma	B7-H3
NCT04185038 (Phase I)	Ventricular delivery of B7-H3-redirected CAR-Ts	70	December 11, 2019 - May 2041	Central nervous system tumors	B7-H3
NCT03500991 (Phase I)	Delivery of HER2-redirected CAR-Ts into the ventricular system or tumor resection cavity	48	July 26, 2018 - July 26, 2039	R/R pediatric central nervous system tumors	HER2
NCT03638167 (Phase I)	Delivery of EGFR806-redirected CAR-Ts into the ventricular system or tumor cavity	36	March 19, 2019 - March 2040	R/R pediatric central nervous system tumors	EGFR806
NCT05103631 (Phase I)	Transgenic expression of IL-15 by CAR-Ts as an attempt to prolong their persistence in the circulation	27	June 17, 2021 - December 2039	Liver cell carcinoma	GPC3
NCT04684563 (Phase I)	CD19-redirected CAR-Ts engineered to express human IL-18	30	May 6, 2021 - May 2036	Non-Hodgkin lymphoma, chronic lymphocytic leukemia	CD19
NCT03085173 (Phase I)	CD19-redirected CAR-Ts engineered to express the co-stimulatory ligand 4-1BBL	39	March 15, 2017 - March 2022	R/R chronic lymphocytic leukemia	CD19
NCT03774654 (Phase I)	CD19-redirected CAR-NKT cells	48	June 22, 2020 - March 2035	R/R B-cell malignancies	CD19
NCT03294954 (Phase I)	GD2-redirected CAR-NKT cells	24	January 18, 2018 - August 10, 2034	Neuroblastoma	GD2
NCT04814004 (Phase I)	CD19-redirected CAR-iNKT cells	20	March 19, 2021 - April 1, 2024	R/R B-cell malignancies	CD19
NCT04702841 (Early Phase I)	CD7-redirected CAR γδ T cells	8	June 3, 2020 - December 2022	R/R T-cell malignancies	CD7

IL, interleukin; iNKT, invariant natural killer T cells; NKT, natural killer T cells; R/R, relapsed/refractory.

## 4 Strategies for Overcoming the Hurdles of Solid Tumor CAR-T Therapy

### 4.1 Boosting Vaccines for Overcoming Insufficient CAR-T Stimulation

The robust proliferation signals provided to CAR-Ts by the antigens they are redirected against mainly contribute to the persistence of CAR-Ts; therefore, the target antigen-expressing tumor cells play the role of APCs (73, 74). In hematologic malignancies, these target cells are easily accessible to CAR-Ts but in solid tumors, it is a whole different scenario due to the TME. Therefore, the population of CAR-Ts decreases quickly since there is no proliferation signals provided to them (36, 75, 76). Recently, Reinhard and collaborators developed a nanoparticle-based RNA vaccine for the delivery of the native-conformation CAR target antigen to the APCs of the lymphoid compartments to augment the expansion rate and tumoricidal efficacy of CAR-Ts *in vivo* (77). Their results indicated that this combination strategy is capable of eliminating large tumors in animal models even with doses of CAR-Ts considered below common therapeutic doses (77). Another vaccine-boosting strategy has explored the use of amphiphile CAR target ligands that migrate to lymph nodes following administration and then position themselves into the bilayer membrane of APCs, thus creating an environment for CAR-T priming (78). According to the results observed in various immunocompetent animal tumor models, this vaccine platform might mediate improved antitumor capacity through amplifying CAR-T expansion and donor cell polyfunctionality (78).

Another study has taken a different trajectory by engineering a nanoemulsion-based vaccine [that selectively targets and activates cross-presenting dendritic cells (DCs)] with full-length recombinant ovalbumin to selectively prime DCs to present ovalbumin to CAR-Ts that are genetically manipulated to surface-express transgenic TCRs specific for ovalbumin (79). Alongside favorable *in vitro* results, the investigators also reported augmented tumor-site trafficking and enhanced proliferation of CAR-Ts which strongly correlated with tumor elimination and durable responses in immunocompetent animal models (79). According to another study, Wu et al. generated an epidermal growth factor receptor pathway substrate 8 DC vaccine (Eps8-DC) and reported that this DC vaccination strategy has beneficial effects on CD19-redirected CAR-Ts including the induction of central memory phenotype development, diminishing activation-induced cell death, augmenting proliferation potential, and amplifying cytokine secretion and tumoricidal capacity (80).

The therapeutic benefits of combining adoptive T-cell therapy with cytomegalovirus-based vaccines have also been evident as this combination was effective in delaying the growth of solid skin tumors in a study by Grenier and colleagues (81). One study has developed GD2-redirected varicella-zoster virus-redirected CAR-Ts (GD2-VZV-CAR-T) for the induction of *in vivo* expansion and persistence of which VZV vaccination can be applied (NCT01953900). Despite the possibilities of these CAR-T growing exhausted after tumor cell antigen engagement, vaccination has been effective in partially reversing the immunoinhibitory effects of the TME *via* TCR stimulation (82). Additionally, Slaney et al. engineered T cells to express both a HER2-redirected CAR and a gp100-specific TCR and combined this product with recombinant gp100-expressing vaccinia virus to target large-sized tumors (including liver and breast tumors) (83). Despite negligible neurotoxicity due to the expression of the target antigen by healthy tissues, this combination therapy strategy was able to mediate durable complete remission (CR) in immunocompetent animals with large-sized tumors owing to vaccine-induced pronounced proliferation and facilitated tumor-site colonization of the engineered T cells (83). Overall, based on these preclinical findings, it might be concluded that various types of vaccines can be applied for improving the therapeutic efficacy of CAR-Ts; however, clinical studies are warranted to further answer the remaining questions in this field.

### 4.2 Strategies for Increasing Poor CAR-T Infiltration Rate

One of the obstacles that weakens the clinical responses of CAR-T therapy in solid tumors is the poor intratumoral CAR-T persistence and trafficking, which can be partly influenced by the cognate receptors on T cells. Recently, attempts have been made to increase the antitumor effects of CAR-Ts through the integration of chemokine receptors into the CAR design (84–86). Moreover, in solid tumors, CAR-T functionality may be deteriorated due to the immunosuppressive characteristics of the TME (87). Such characteristics include the expression of inhibitory ligands such as PD-L1, inhibitory mechanisms applied by regulatory cells, malignancy-associated metabolic dysregulation through various enzymes such as indoleamine-2,3-dioxygenase (IDO), and the presence and suppressive effects of other inhibitory factors such as TGF-β (87). In this section, we review studies that have made elaborate attempts for improving the antitumor functionality and infiltration rate of CAR-Ts in solid tumors.

#### 4.2.1 Engineered Expression of Cytokines/Chemokines or Their Receptors

The tumor-mediated overexpression and secretion of the chemokine IL-8 (CXCL8) have been utilized as leverage to maximize the tumoricidal effects of CAR-Ts in solid tumors by increasing the intratumoral trafficking of these cells (84). In detail, genetic modification of CAR-Ts for the expression of IL-8 receptors (CXCR1 or CXCR2) and the utilization of the tumor-secreted IL-8 to usher the IL-8 receptor-modified CAR-Ts (IL-8R-CAR-Ts) to the tumor foci results in enhanced antitumor activity (84, 86). It has been evident that not only IL-8R modification does not impinge on the ability of the modified T cells to recognize tumor cells, but it also increases *in vitro* T-cell chemotaxis in the presence of the chemokine (84). Animal studies have indicated that IL-8R-CAR-Ts exhibit superior characteristics in comparison to conventional CAR-Ts (84). In a particular case, deferred migration and low intratumoral presence of CD70-redirected CAR-Ts (CD70-CAR-Ts) have been known to contribute to the formation of an immunosuppressive TME that leads to potential tumor relapse despite the consistent tumor cell CD70 expression and peripheral persistence of the CD70-CAR-Ts (84). It has been evident that reversal of tumor-induced immunosuppression, inhibition of tumor growth, and maximized antitumor responses are achievable through the natural or ionizing radiation-induced secretion of IL-8 that facilitates the intratumoral trafficking of IL-8 receptor-modified CD70-CAR-Ts (IL-8R-CD70-CAR-Ts) (84, 88). According to Jin and collaborators, IL-8R-CD70-CAR-Ts exhibited pronounced presence and persistence in tumor milieus inducing complete tumor elimination and prolonged immunologic memory in glioblastoma, ovarian, and pancreatic cancer animal models (84). Additionally, it has been evident that engineered co-expression of CAR and C-C chemokine receptor 4 (CCR4) by effector T cells can result in enhanced tumor-site trafficking and tumoricidal cytotoxicity (89). In detail, CCR4 is the specific receptor of C-C motif chemokine ligand 17 (CCL17) and C-C motif chemokine ligand 22 (CCL22) which are secreted by DCs and macrophages for the chemotaxis of Tregs and type 2 T helper (Th2) cells (89). Tumor cells have been known to produce these chemokines for the formation of an immunosuppressed TME, a phenomenon that now has been known to be a double-edged sword against them (89). Furthermore, CAR-Ts engineered to express the chemokine receptor CCR2b, which is the receptor of C-C motif chemokine ligand 2 (CCL2), have exhibited more than ten-fold enhanced migration towards CCL2-secreting tumor cells alongside amplified antitumor capacity in comparison with conventional CAR-Ts (90, 91). Such findings highlight the potential of these strategies for overcoming the inadequate tumor localization of CAR-Ts (90, 91). According to another study, intravenous (IV) administration of CXCL11-modified oncolytic vaccinia virus (CXCL11-OVV) in mouse tumor models can be employed to modify tumor cells to secrete CXCL11, thus increasing the intratumoral concentration of CXCL11 (92). This phenomenon has been known to mediate a more pronounced tumor rejection by increasing the intratumoral accumulation of CAR-Ts (92).

#### 4.2.2 Glycoengineering

The vessels of bone marrow and most tumors express endothelial molecules such as the lectin E-selectin which can be utilized as leverage for the extravasation CAR-Ts (93). Sialyl Lewis X (sLeX) acts as the cognate ligand of E-selectin, which is not expressed by T cells (hence not expressed by CAR-Ts either) (93). On the other hand, CAR-Ts exhibit a high profile of sialylated type 2 lactosamine expression that can be doctored to sLeX through α (1, 3)-fucosylation but since culture-dependent expansion downregulates the endogenous fucosylation pathway, engineered fucosylation using α (1, 3)-fucosyltransferase and GDP-fucose can be chosen to carry out this mission (93). According to Mondal and colleagues, the glycoengineered sLeX expression on CAR-Ts increases their infiltration capacity (around 10-fold) into the marrow of mouse models, compared to conventional CAR-Ts, thereby allowing better tissue colonization and obviating the need for high-dose adoptive cell administrations (93). These findings introduce cell surface glycoengineering as an as-is translatable approach for enhancing the homing of CAR-Ts in tissues with E-selectin-expressing endothelial cells. However, more substantiated findings may be required in this regard for such conclusions.

#### 4.2.3 Protein Kinase A (PKA) Blockade

Resistance of CAR-Ts to immunosuppressive molecules such as adenosine or prostaglandin E2 can be also implemented to augment CAR-T efficacy and facilitate their tumor-site trafficking (94). In detail, adenosine or prostaglandin E2 activate PKA which results in its association with *ezrin* leading to T-cell hypofunction (94, 95). In 2016, Newick et al. developed CAR-Ts expressing an inhibitory peptide designated as RAID (regulatory subunit I anchoring disruptor) (94). These researchers demonstrated that RAID nullifies the inhibitory impacts of PKA on TCR activation by disrupting its association with ezrin (94). In comparison with conventional CAR-Ts, RAID-expressing CAR-Ts exhibited better tumoricidal efficacy owing to their better matrix adhesion and augmented trafficking in response to CXCL10 (94). Based on the *in vitro* and *in vivo* findings, Newick et al. suggested that this tactic might have clinical application after passing the necessary evaluations (94).

#### 4.2.4 Photothermal Therapy

Regional hyperthermia of tumors may contribute to the recruitment of bystander immune cells by antigen spreading, diminishing the pressure of interstitial fluids, and disrupting the structural compaction of tumor tissues (96). It has recently been evident that mild hyperthermia of tumor sites can broaden the therapeutic reach of CAR-Ts into the milieu of solid tumors; therefore enhancing their tumoricidal efficacy (96). In detail, Chen et al. reported that the chondroitin sulfate proteoglycan-4 (CSPG4)-redirected CAR-Ts administered into melanoma NOD scid gamma (NSG) mouse models, which were established using the human melanoma cell line WM115, exhibited enhanced tumoricidal activity after tumor ablation using photothermal therapy (96). In addition to this study, Miller et al. evaluated the applicability of synthetic gene switches that mediate the expression of transgenes in specific responses to mild temperature increases (around 40 to 42 °C) (97). These researchers demonstrated that *in vitro* thermal therapy in primary human T cells led to a considerably higher expression level of a reporter transgene without any negative effects on the expansion, migration, and antitumor activity of the T cells (97). Moreover, these researchers also indicated that expression of an IL superatagonist or T-cell-redirecting bispecific antibodies (TRBAs) induced by intratumoral photothermal therapy improved tumoricidal responses and reduced antigen escape in mouse models after the systemic administration of CAR-Ts (97). However, such ideas cannot be considered as general solid tumor CAR-T therapy solutions until more in-depth preclinical and clinical assessments are carried out.

#### 4.2.5 Application of Docetaxel, Antiangiogenic Drugs, or NEO100

It has been elucidated that docetaxel can also amplify the antitumor activity of CAR-Ts by expanding their action zone into the TME (98). The presence of docetaxel induces a higher expression profile of high mobility group box 1 (HMGB1) from tumor cells which in turn induces the expression of CXCL11 through NF-κB activation (98). CXCL11 upregulation strongly facilitates CAR-T infiltration into the TME and correlates with better therapeutic benefits as reported by Gao and colleagues (98).

Moreover, transient remodeling of tumor-associated vasculature using antiangiogenic molecules may further enhance the therapeutic impact of cancer immunotherapy by expanding the extravasation rate of CAR-Ts into the TME (99, 100). In a neuroblastoma preclinical model, it has been demonstrated that *Bevacizumab* (a clinically approved anti-VEGF-A mAb) can increase the infiltration and tumoricidal capacity of GD2-redirected CAR-Ts (99, 101). Furthermore, CAR-T-mediated secretion of interferon-γ also contributed to the induction of neuroblastoma cell-mediated CXCL10 expression (99). Another study has also reported augmented tumor-site CAR-T accumulation by the help of vascular disrupting agents (100). According to Deng and colleagues, combretastatin A-4 phosphate (CA4P) has been effective in expanding the therapeutic reach of CAR-Ts by enhancing their tumor colonization in preclinical mouse models of ovarian and colon cancer (100).

Furthermore, blood-brain barrier (BBB) impermeability is also considered an obstacle in the case of central nervous system (CNS) tumor CAR-T therapy (102). Intraarterial (IA) administration of NEO100 (which is the purified form of perillyl alcohol (POH)) has been known to safely and reversibly permeabilize the BBB in mice by importing the tight junction-associated endothelial membrane proteins into the cytoplasm (102). This permeabilization paves the way for a more efficient CNS presence of CAR-Ts resulting in more pronounced tumoricidal responses, according to Wang et al. (102).

#### 4.2.6 Regional Delivery of CAR-Ts

Regional delivery of CAR-Ts has recently emerged as a potent strategy for increasing the therapeutic reach of CAR-T therapy in solid tumors (103–109). In particular, Katz et al. reported that regional intraperitoneal (IP) CAR-T delivery leads to a superior tumoricidal capacity against carcinoembryonic antigen (CEA)-positive tumors of colorectal cancer that have metastasized to the peritoneum, compared with systemic administration (105). Moreover, these researchers also added that CAR-Ts delivered regionally to the peritoneum exhibited a rise in effector memory phenotype over time alongside suppressing tumor progression in distant subcutaneous (SC) regions with the involvement of elevated in-serum IFN-γ levels (105). Moreover, Katz et al. also studied IP CAR-T delivery with suppressor cell targeting therapy since MDSCs, with high levels in immunosuppressive PD-L1 expression, and Tregs are highly populated inside IP tumors (105). They also reported that this method resulted in enhanced antitumor activity and suppression of the peritoneum-metastasized tumors (105). In another experiment, Priceman et al. investigated three delivery routes including intravenous, local intratumoral, or regional intraventricular for HER2-redirected CAR-T administration into human xenograft mouse models with leptomeningeal disease and brain-metastasized breast cancer (106). These researchers reported that the regional intraventricular delivery of HER2-redirected CAR-Ts resulted in promising therapeutic responses against leptomeningeal disease and brain-metastasized breast cancer in human xenograft mouse models (106).

IV and regional delivery of HER2-redirected CAR-Ts have also been compared for the elimination of medulloblastoma in animal models (104). It has been evident that the induction of durable antitumor responses *via* the IV route requires a higher dosing scheme in comparison to the regional delivery of CAR-Ts (104). Moreover, it has been concluded that intraventricular delivery of HER2-redirected CAR-Ts in non-human primates does not contribute to the emergence of systemic toxicities and can be considered well-tolerated (104). Based on these outcomes, researchers have proposed that direct delivery of HER2-redirected CAR-Ts into the cerebrospinal fluid (CSF) can be investigated in clinical trials with recurring medulloblastoma patients enrolled (104). Besides the already mentioned investigations, another recent study has also validated the feasibility and safety of intrathecal delivery of EPHA2-, HER2-, and IL-13 receptor α2-redirected CAR-Ts in xenograft mouse models (108). The researchers indicated that this CAR-T administration route is potentially efficacious for the regression of posterior fossa group A ependymomas and medulloblastoma with or without concurrent *azacitidine* administration (a chemical analog of *cytidine*) (108).

Other researchers have also reported that locoregional delivery of B7-H3-redirected CAR-Ts through intracerebroventricular or intratumoral administration has also been correlated with lower levels of systemic inflammatory cytokines and pronounced tumoricidal responses in xenograft mouse models of atypical teratoid/rhabdoid tumors in comparison with intravenously infused B7-H3-redirected CAR-Ts (103). Of note, an ongoing Phase I clinical trial (NCT03500991) is currently investigating the locoregional delivery of HER2-redirected CAR-Ts in children and young adults with R/R tumors of CNS (110). According to a recent report of this trial by Vitanza et al., HER2-redirected CAR-Ts were delivered into the tumor cavity or the ventricular system using a CNS catheter (110). Moreover, no dose-limiting adverse events were reported in the enrolled patients (110). Based on the findings, local CNS immune responses were documented which were associated with significant CCL2 and CXCL10 levels in the CSF (110). Overall, such clinical data propose that repeated delivery of CAR-Ts into the CNS of children and young adults with R/R tumors of CNS might be well-tolerated and result in local CNS immune reactions (110). However, broader clinical findings in this regard can further validate the strategies discussed in this section.

### 4.3 Overcoming Low Target Antigen Density or Lack of Definitive Target Antigens Through Induced Expression of CAR Targets in Tumor Cells

CAR-T therapy of solid tumors suffers from poor clinical responses partly due to the lack of definitive or highly expressed tumor antigens. Recently, various strategies have been developed to break the reliance of solid tumor CAR-T therapy on endogenous tumor antigens through the induced expression of CAR target antigens in tumor cells (111–113). A recent study by Park and colleagues has used an oncolytic vaccinia virus equipped with the nucleotide sequence of a truncated form of CD19 (truncCD19OV) to infect tumor cells for the surface expression of truncCD19 enabling their targeting with CD19-redirected CAR-Ts (113). *In vitro* and *in vivo* results indicated that the infected tumor cells exhibited considerable cytolytic sensitivity to CD19-redirected CAR-Ts (113). Furthermore, alongside the induction of endogenous antitumor effects by truncCD19OV, its continuous release from the tumor cells cytolyzed by CD19-redirected CAR-Ts contributed to the promotion of the antigen expression by other tumor cells (113). In 2020, Tang et al. developed recombinant adenoviruses harboring tumor-specific promoters that drive the expression of CD19 in tumor cells rendering them susceptible to CD19-redirected CAR-Ts and later engineered the viruses with replication capability (112). These researchers demonstrated that this oncolytic antigen-labeling strategy was capable of inducing tumor rejection through the formation of cytotoxic immunological synapses between tumor cells and CD19-redirected CAR-Ts in animal models, thus increasing their survival rate (112).

**Figure 3:**
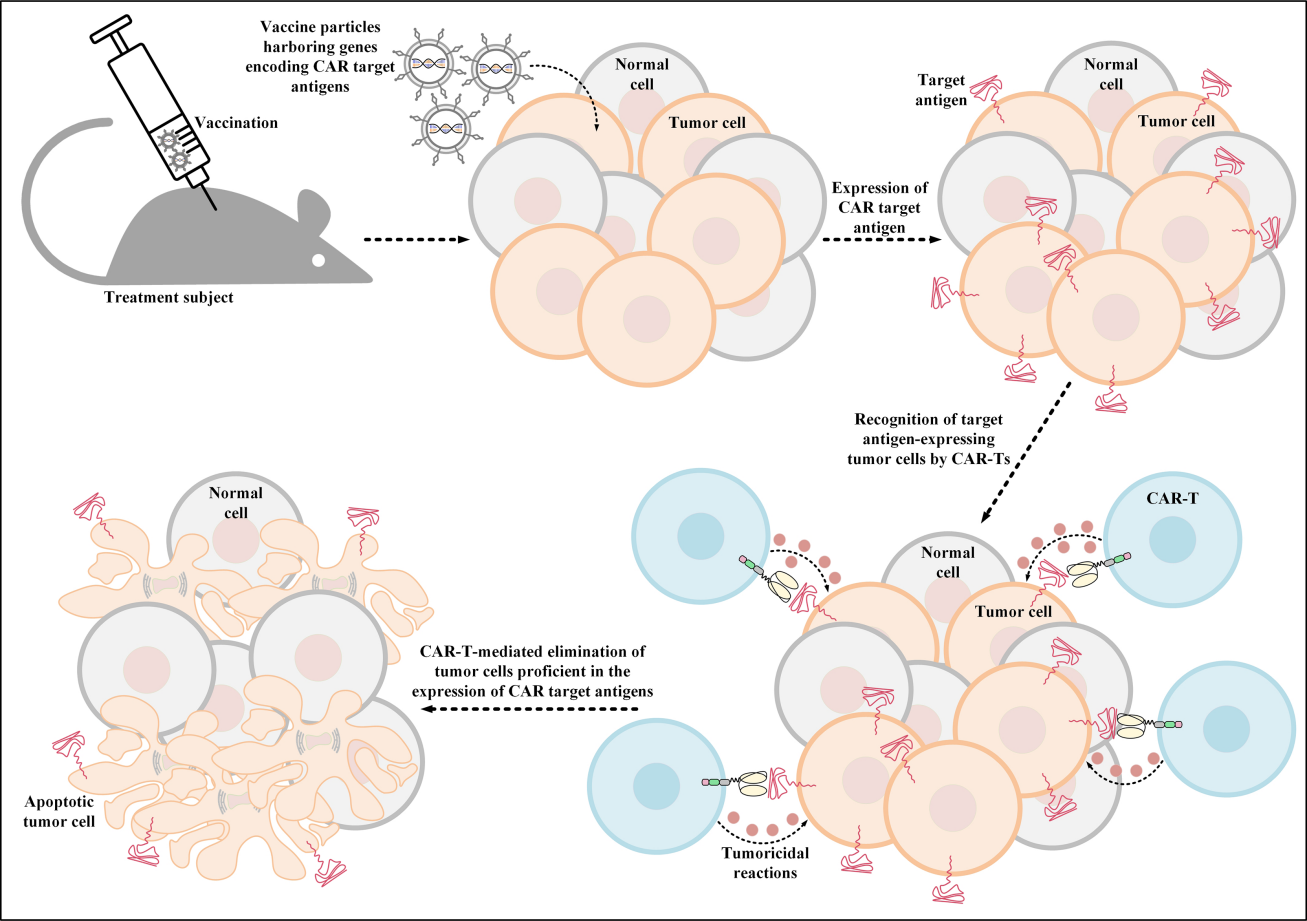
Oncolytic vaccinia virus-mediated induction of CAR target antigen expression. This type of induced CAR target antigen expression in tumor cells results in efficient recognition and elimination of target antigen-expressing tumor cells by CAR-Ts. CAR; chimeric antigen receptor.

Additionally, another investigation has also developed thymidine kinase-disrupted oncolytic vaccinia viruses to selectively infect cancer cells for CD19 expression (111). In detail, Aalipour et al. demonstrated that utilization of this strategy correlated with considerably prolonged survival of immunocompetent animal models, which was a result of CD19-redirected CAR-T-mediated tumor elimination (**Figure 3**) (111). These novel findings accentuate the importance and feasibility of these strategies for tackling the antigen-related obstacles of solid tumor CAR-T therapy; however, more in-depth investigations are required in this field to better assess the applicability of these tactics.

### 4.4 Overcoming the Immunosuppressive TME

Cytokines that are capable of boosting the tumoricidal activity of CAR-Ts can be utilized as leverage for overcoming the immunosuppressive nature of TME that contributes to the functional exhaustion and metabolic starvation of CAR-Ts (42, 114, 115). However, constitutive secretion of transgenic cytokines by CAR-Ts and the presence of their corresponding receptors on bystander immune cells, such as T cells and NK cells, contribute to the emergence of life-threatening adverse events to take which under control requires the implementation of safety switches (which are comprehensively discussed elsewhere) (46, 116–118).

Recently, it has been demonstrated that STAT3 signaling can improve the tumoricidal activity of CAR-Ts leading to more favorable antitumor responses (119, 120). In particular, high concentrations of IL-23, achieved through the administration of its recombinant form or its secretion by gene-modified tumor cells, have been associated with antitumor impacts (121, 122). Moreover, this two-subunit STAT3-activating cytokine (composed of IL-23α p19 and IL-12β p40 subunits) has been known to act as a pro-proliferative and a tumoricidal effectivity-enhancing cytokine for memory T cells and T helper 17 (Th_17_) cells that express its cognate receptor, IL-23R (123–128). Since IL23-R and IL-23α p19 are upregulated upon TCR antigen engagement and the IL-12β p40 subunit is not, Ma et al. demonstrated that engineered expression of the p40 subunit by T cells (p40-Td cells) can lead to their autocrine IL-23 signaling-induced selective proliferation, expansion, and survival upon activation (114). These researchers reported that p40-Td CAR-Ts exhibited augmented tumoricidal functionality characterized by the upregulation and downregulation of granzyme B and PD-1, respectively, compared with conventional CAR-Ts (114). The researchers also added that in mouse models of solid tumors, p40-Td CAR-Ts exhibited superior antitumor activity and decreased adverse complications in comparison with conventional CAR-Ts and IL-18- or IL-15-expressing CAR-Ts, respectively (114). The engineered expression of the p40 subunit can sufficiently mediate the expression and secretion of functional IL-23 exclusively by activated T cells in response to their activation within the TME since IL-23 is only assembled when both of the subunits are upregulated (114). This creates an autocrine action mode for the secreted IL-23 that provides a high-level regulation for the IL-23–IL-23R pathway (114, 121, 122). This prevents cytokine spread and bystander immune cells to be affected by the cytokine secretion while minimizing the side effects observed in other cytokine expression or administration strategies (114, 121, 122).

Additionally, IL-2 is also considered as a cytokine necessary for T-cell expansion, function, and survival modulation (129). However, the pleiotropic characteristics of IL-2 have overshadowed its broad application due to the emergence of toxicities (129). High-dose IL-2 is used in the treatment of patients with renal cell carcinoma and melanoma (130, 131). However, appropriate clinical settings are required for a safe IL-2 utilization and the prevention of treatment-related mortality (130, 131). IL-2 administration-related toxicities can occur in multiple organs including the heart, lungs, and CNS (130, 131). Capillary leak syndrome is known as the most frequent IL-2 administration-related toxicity (130, 131). This toxicity leads to a hypovolemic condition and massive plasma leakage from blood vessels into the extravascular space (130, 132). Capillary leak syndrome can lead to common clinical conditions such as oliguria, ischemia, and confusion (130, 132). Even though high-dose IL-2 administration can be accompanied by such severe and life-threatening toxicities, safe and effective high-dose IL-2 administration can be achieved by using highly experienced healthcare professionals and toxicity prevention and controlling approaches (130, 131). A recent study has elaborately designed mutant forms of IL-2 (orthogonal IL-2) and its relative receptor, IL-2R (orthogonal IL-2R), that do not interact with their respective native counterparts, but in the meantime are capable of triggering native IL-2 downstream signaling cascades after specific interaction with each other (129). At-will potentiation of T cells engineered to express the orthogonal IL-2R has been achieved in preclinical animal models by the administration of orthogonal IL-2 without mediating considerable adverse events or complications (129). Such findings highlight the potential of this engineering twist and propose it as a translatable approach in CAR-T therapy; however, careful preclinical and clinical experiments are required in this regard (129).

Recently, one study has discovered that engineered expression of the native form erythropoietin receptor (EpoR) or its truncated form (tEpoR) on the surface of CAR-Ts endows them with the ability to tune up their expansion, survival, and proliferation rate in response to erythropoietin (133). It has been evident that tEpoR exhibits supervisor characteristics, in comparison to EpoR, in terms of T-cell expansion, proliferation, and survival stimulation (133). Vinanica et al. generated tEpoR-expressing CD19-redirected CAR-Ts (tEpoR-CAR-Ts) and reported that these cells demonstrated pronounced *ex vivo* expansion and *in vitro* tumoricidal capacity against leukemic cells in the presence of erythropoietin (133). These results confirmed that the expression of either receptor does not negatively impact the functionality of the other (133). Furthermore, tEpoR-CAR-Ts have been known to require lower cell dosing, in comparison with their conventional counterparts, since physiologic levels of erythropoietin would simply suffice to expand tEpoR-CAR-Ts, thus eliciting an accentuated tumoricidal response in mouse models (133). Of note, erythropoietin and ruxolitinib can be utilized to amplify the effector function of tEpoR-CAR-Ts or to diminish unwanted complications, respectively (133).

## 5 Strategies for Amplifying Antitumor Responses Though the Induction of Bystander Antitumor Effects

As mentioned earlier, solid tumor CAR-T therapy suffers from inconsistent clinical responses and tumor escape that are partly resultant from heterogeneous tumor antigen expression and tumors down-regulating or not expressing the antigen targeted by single-antigen targeting CAR-Ts (41). In such cases, the induction of endogenous immune response against target antigens other than those recognized by the adoptively transferred CAR-Ts can be beneficial. This approach can expand tumor-redirected immune responses *via* the recruitment of a wide range of endogenous T cells infiltrated in the tumor site resulting in more probable prevention of tumor antigen escape (134–137). In the upcoming section, we review strategies that can be exploited to trigger bystander endogenous antitumor responses concurrent with the tumoricidal effects of CAR-Ts.

### 5.1 TRBA-Secreting CAR-Ts

**Figure 4:**
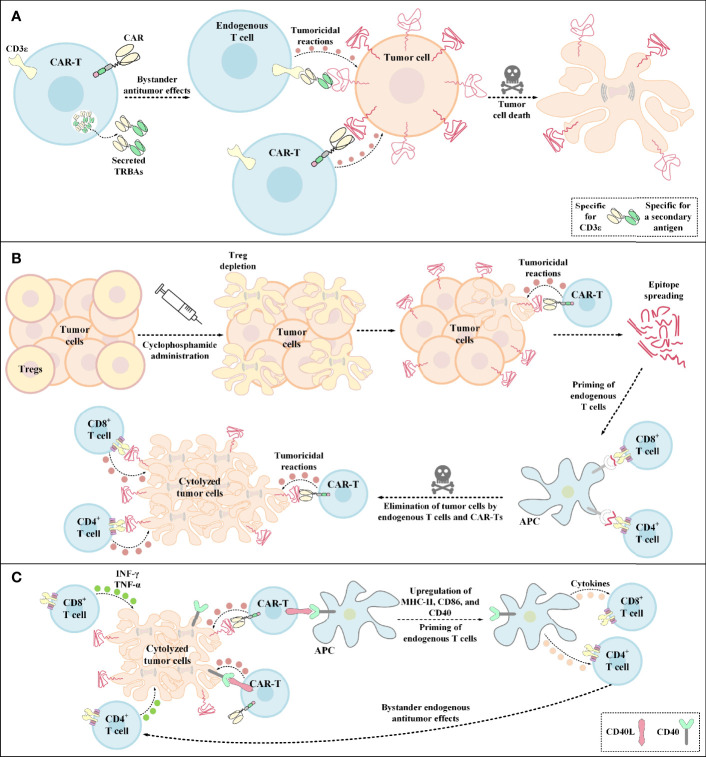
Bystander antitumor effect induction by TRBA-secreting CAR-Ts, cyclophosphamide administration, and CD40L-expressing CAR-Ts. **(A)** TRBA-secreting CAR-Ts. TRBAs are made of two scFvs fused *via* a linker peptide. One of these scFvs targets CD3 (present on the surface of endogenous T cells) and the other one targets a TAA or TSA of interest against which endogenous T-cell responses are intended to be redirected. TRBA-secreting CAR-Ts secrete these bispecific T-cell-redirecting antibodies which results in endogenous T-cell-mediated antitumor reactions against malignant cells alongside CAR-T-mediated tumoricidal responses enabling a more effective tumor cell elimination. **(B)** Cyclophosphamide administration. Cyclophosphamide administration mediates Treg depletion and enables a more efficient CAR-T engagement with its target antigen and the subsequent CAR-T-mediated tumoricidal reactions. Additionally, upon epitope spreading, APCs uptake the released peptide antigens and present them to CD4^+^ T cells and CD8^+^ T cells. This mechanism leads to the priming of endogenous T cells and the subsequent elimination of tumor cells through bystander antitumor effects mediated by these endogenous cells. **(C)** The mechanism of action of CD40L^+^ CAR-Ts. CD40L^+^ CAR-Ts can mediate tumor cell cytolysis through both their CAR and their CD40L interacting with the CAR target antigen and CD40 on the surface of tumor cells, respectively. Additionally, CD40L^+^ CAR-Ts mediate DC licensing as indicated by the upregulated level of CD40, CD86, and MHC-II. These APCs in turn recruit other immune effector cells such as endogenous T cells. INF-γ and TNF-α secretion by the recruited endogenous CD4^+^ T cells and CD8^+^ T cells also result in tumor cell cytolysis. APC, antigen-presenting cells; CAR, chimeric antigen receptor; CD40L, CD40 ligand; INF-γ, interferon γ; MHC-II, major histocompatibility complex class II; TNF-α, tumor necrosis factor α; TRBAs, T-cell-redirecting bispecific antibodies; Treg, regulatory T cell.

In the case of glioblastoma (GBM), it has been demonstrated that a single-dose administration of EGFRvIII-redirected CAR-Ts, a GBM-specific tumor antigen, can contribute to the mediation of antigen loss and adaptive resistance in recurrent GBM patients with the tumor retaining high levels of wild-type EGFR expression (36). Recently, an elaborate strategy has been developed to overcome this issue by the incorporation of CARs and TRBAs into a single gene-manipulated T-cell product called CART.TRBA (41). In this strategy, a bicistronic construct encodes an EGFRvIII-specific CAR and TRBAs against the wild-type EGFR; thereby obviating the need for multiple transgene insertions and reducing the risks of insertional mutagenesis (41, 138, 139). TRBAs secreted by CART.TRBA cells not only redirect CAR-Ts to the respective tumor sites, but they can also act in a paracrine manner by recruiting bystander T cells against the tumor (**Figure 4A**) (41). Alongside minimizing the effects of EGFRvIII antigen loss, local TRBA secretion also reduces the risk of on-target toxicity associated with systemic administration of TRBAs (41). Moreover, CART.TRBA-mediated local TRBA secretion also erases the scenario for repeated TRBA infusions since TRBAs are sometimes subject to rapid renal clearance due to their low molecular weight (41). Furthermore, the desired differentiation and phenotype of the T cells can also be simply achieved by their simultaneous redirection using CARs and TRBAs (41). Not only may the CART.TRBA platform serve as a therapeutic option for GBM, but it may also have potent applicability for other types of solid cancers with heterogeneous EGFRvIII expression (including breast cancer, medulloblastoma, and ovarian carcinoma) (41, 140–142). Additionally, this approach may also be utilized for other types of antigen loss- or escape-associated tumors by targeting different combinations of tumor antigens (41).

Other studies have also proposed strategies for tackling antigen heterogeneity using TRBA-expressing oncolytic viruses (143, 144). According to a study by Wing et al., folate receptor-alpha-redirected CAR-Ts (FR-α-CAR-Ts) failed to achieve complete tumor elimination in xenograft tumors due to antigen loss (143). In contrast, these researchers reported that enhanced tumoricidal responses and prolonged survival were observed when anti-EGFR TRBA-expressing oncolytic viruses were combined with FR-α-CAR-Ts (143). The secreted TRBAs redirected FR-α-CAR-Ts and bystander T cells towards the tumor alongside favoring their activation, expansion, cytokine secretion, and tumoricidal effects (143).

Recently, Porter et al. expanded the use of oncolytic viruses for the production of immune checkpoint blockers (anti-PD-L1 antibodies), TRBAs (specific for the variant 6 of CD44), and an immunostimulatory cytokine (namely IL-12) by incorporating the nucleotide sequence of the TRBA into an oncolytic-helper binary adenovirus to simultaneously tackle antigen heterogeneity and TME-mediated immunosuppression (144). It is encouraging to conclude that these researchers reported that the combination of the resultant trio adenovirus with HER2-redirected CAR-Ts resulted in more reliable tumor rejection and survival through the engagement of non-specific immune cells, while compared with the respective monotherapies (144). Such strategies can be applied to overcome the limitation of rapid TRBA renal clearance and obviate the need for its sequential administrations.

### 5.2 Fms-Like Tyrosine Kinase 3 Ligand (Flt3L) Expression

Recently, it has been demonstrated that induction of pronounced antitumor responses and expanded epitope spreading is achievable through the combination of agonistic anti-4-1BB antibody, poly (I:C), and T cells genetically modified to express Flt3L (145). Flt3L is a DC growth factor and poly (I:C) is a Toll-like receptor 3 agonist that contributes to DC maturation, interferon secretion, and augmentation of T-cell stimulation capability (145, 146). As Lai et al. have reported, this combination therapy is capable of tackling tumor escape variants because of its ability to drive antitumor responses against a broad spectrum of tumor antigens through vast epitope spreading (145). Such antigens include those that are not programmed to be targeted by a particular ACT (145). These researchers have also proposed that utilizing endogenous DCs serves as a potent tactic for tackling the limitation of antigen-negative tumor escape after specific TCR or CAR-T therapies (145).

### 5.3 Cyclophosphamide Administration

Pretreatment with cyclophosphamide below the lymphodepleting dose has been recently known to induce bystander antitumor effects most likely through endogenous CD8^+^ T cells (147). It has also been evident that this strategy can mediate TME immunomodulation through the depletion of Tregs (**Figure 4B**) (147). In a particular case, Klampatsa and colleagues reported that mesothelin-redirected CAR-Ts were unable to fully eradicate tumor cells in mouse models when only a small proportion of the tumor cells grew deficient in the expression of the targeted antigen (147). These researchers indicated that co-infusion of IDO inhibitor or PD-1-, CTLA-4-, or TGF-β-specific antibodies did not mediate any bystander antitumor effects (147). In contrast, it was evident that pretreatment with low-dose cyclophosphamide was effective in eliminating a larger proportion of mesothelin-negative tumor cells by inducing bystander antitumor effects (147). It was also reported that these bystander antitumor effects were CD8^+^ T lymphocyte-dependent, rather than DC-dependent (147).

### 5.4 CD40 Ligand (CD40L)-Expressing CAR-Ts

Constitutive expression of CD40L by genetic engineering of CAR-Ts (CD40L^+^ CAR-Ts) can be another strategy for tackling antigen loss-related immune escape of tumor cells by operating through direct and indirect tumoricidal responses (148, 149). The direct antitumor effect is established through the CD40/CD40L interaction between CD40L^+^ CAR-Ts and CD40^+^ tumor cells (148). On the other hand, the indirect effect arises from CD40L^+^ CAR-Ts’ ability for licensing APCs which results in the recruitment, mobilization, and cytokine production of endogenous effector cells that are capable of tumor recognition (**Figure 4C**) (148). According to one study, Curran et al. reported that CD40L^+^ CAR-Ts are also capable of increasing the immunogenicity of CD40^+^ tumor cells by increasing the expression level of HLA molecules, costimulatory molecules, Fas receptor, and adhesion molecules such as CD70 (149). These researchers also added that these CAR-Ts exhibited boosted expansion and production of proinflammatory TH1 cytokines alongside mediating the maturation of monocyte-derived DCs (Mo-DCs) and inducing the production of IL-12 by them (149). Ultimately, Curran et al. proposed that the clinical application of CD40L+ CAR-Ts might lead to improved outcomes in CAR-T therapy of solid tumors (149).

### 5.5 C-C Motif Chemokine Ligand 19 (CCL19)- and IL-7-Secreting CAR-Ts

Additionally, it has been demonstrated that fibroblastic reticular cells mediate lymphoid neogenesis in which peripheral lymphocytes and DCs are recruited (150). CCL19 (which act as a chemo-attractant for T cells and DCs) and IL-7 (which is an essential regulator of T-cell proliferation and survival) are crucial homeostatic cytokines produced by fibroblastic reticular cells during the formation of lymphoid organs (151–154). With this in mind, CAR-Ts engineered to produce CCL19 and IL-7 (CCL19-IL-7 CAR-Ts) can act in a similar fashion as fibroblastic reticular cells in terms of T cell and DC recruitment to the desired tumor sites to help amplify tumoricidal effects (150). In 2018, Adachi et al. reported that CCL19-IL-7 CAR-Ts exhibited superior tumoricidal cytotoxicity, in comparison with conventional CAR-Ts, and that these cells benefited from the collaboration of other immune cells in fighting against tumors (150). Moreover, these researchers concluded that not only simultaneous expression of CAR and other immune-regulatory molecules such as IL-7 and CCL19 results in the augmentation of antitumor responses but also contributes to the activation and memory development of conventional T cells (150).

In a recent study, Goto et al. generated CCL19-IL-7 CAR-Ts and reported that these cells mediated effective tumoricidal responses in mouse models of solid tumors with enhanced tumor-site trafficking and expansion even in the immunosuppressive TME (155). In detail, they reported that human mesothelin-redirected CCL19-IL-7 CAR-Ts completely eliminated orthotopic malignant mesothelioma and prevented disease relapse with proportional antigen loss in mouse models (155). Moreover, it was reported that mesothelin-redirected CCL19-IL-7 CAR-Ts mediated meaningful suppression of tumor outgrowth in mice PDX models of mesothelin-expressing pancreatic cancers as compared with conventional CAR-Ts (155). Additionally, these researchers reported that the administration of CCL19-IL-7 CAR-Ts into mouse models also led to an increase in the number of other tumor-site trafficking immune cells alongside suppressing the expression of exhaustion markers (such as PD-1) on the endogenous T lymphocytes (155). Such findings might suggest the suitability of this strategy for the CAR-T therapy of patients with solid tumors; however, after broader preclinical and clinical investigations are carried out successfully (155).

Similarly, Pang et al. also generated GPC3-redirected CCL19-IL-7 CAR-Ts and reported that these cell exhibited improved proliferation and migration functionality according to *in vitro* assessments and more advanced antitumor activity in hepatocellular carcinoma (HCC) and pancreatic carcinoma cell line-established xenograft models (156). These researchers also reported the early results of an ongoing Phase I clinical trial (NCT03198546) in patients with advanced HCC, pancreatic carcinoma, or ovarian carcinoma who have glypican-3 (GPC3) or mesothelin expression (156). According to this report, the intratumor administration of GPC3-redirected CCL19-IL-7 CAR-Ts into a patient with advanced HCC resulted in complete tumor rejection within 30 days following the therapy (156). Moreover, the intravenous (IV) administration of MSLN-redirected CCL19-IL-7 CAR-Ts into a patient with advanced PC led to complete tumor elimination 240 days after the therapy (156). Such preclinical and clinical data support the effectiveness of this strategy for the improvement of CAR-T therapy in solid tumors and pave the way for more clinical studies with broader patient populations (150, 155–157).

### 5.6 IL-36γ-Secreting CAR-Ts

**Figure 5:**
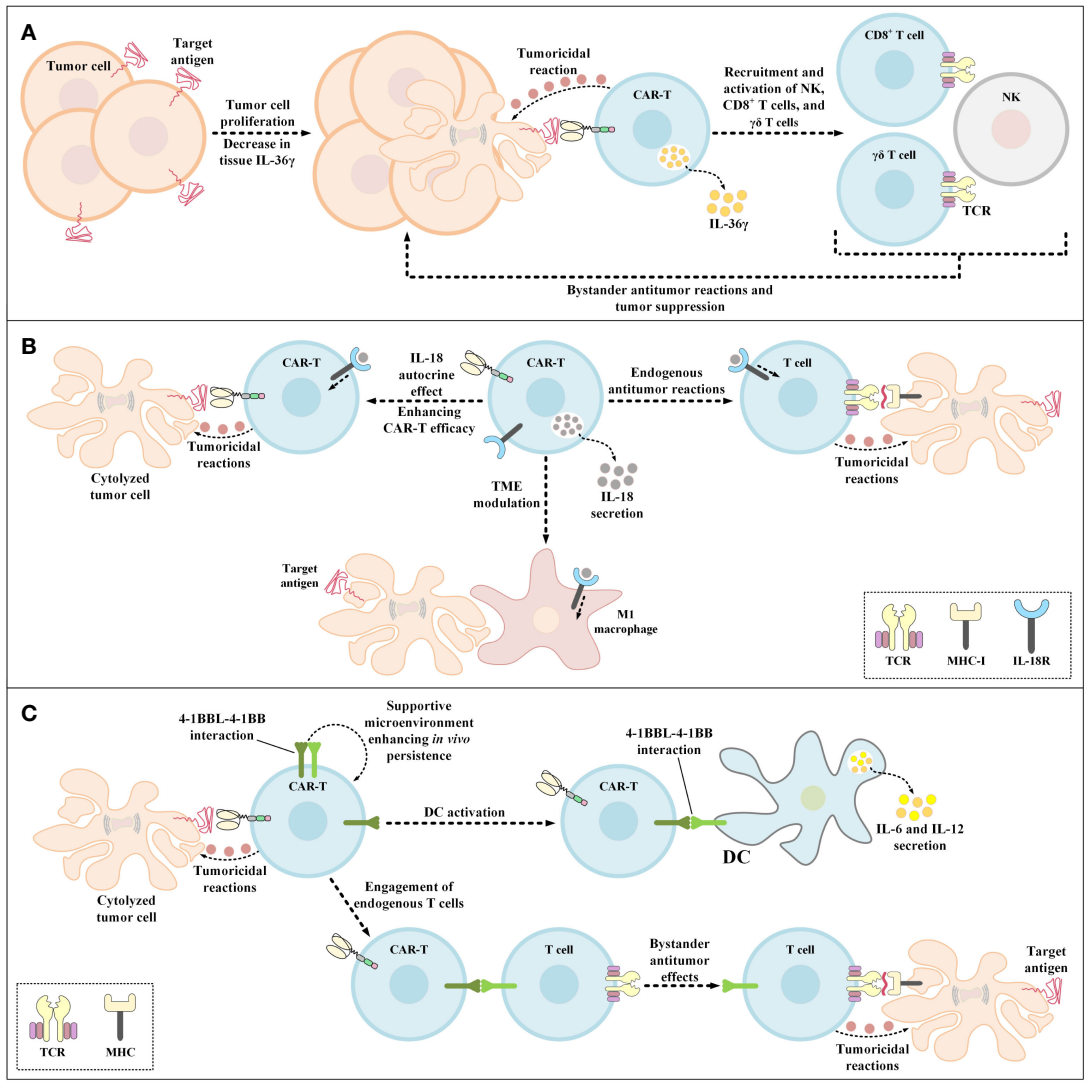
Bystander antitumor effect induction using IL-36γ-secreting CAR-Ts, IL-18-secreting CAR-Ts, and 4-1BBL-expressing CAR-Ts. **(A)** The effects of IL-36γ secreted by IL-36γ-secreting CAR-Ts. IL-36γ secreted by these engineered CAR-Ts results in the recruitment and activation of endogenous immune effector cells, which include type 1 lymphocytes such as CD8^+^ T cells, NK cells, and γδ T cells. These endogenous immune effector cells can mediate bystander antitumor immune reactions resulting in the suppression of tumor cell proliferation. **(B)** The effects of IL-18 secreted by IL-18-secreting CAR-Ts. CAR-T-secreted IL-18 has autocrine effects on the CAR-Ts themselves enhancing the antitumor activity of these cells. In case of endogenous tumoricidal effects, CAR-T-secreted IL-18 recruits endogenous T cells to the tumor sites and triggers their bystander antitumor reactions. Moreover, CAR-T-secreted IL-18 also modulates the TME and recruits M1 macrophages to the tumor site which results in M1 macrophage-mediated tumor cell cytolysis. **(C)** The beneficial effects of 4-1BBL expression by CAR-Ts. 4-1BBL-expressing CAR-Ts demonstrate enhanced functionality in comparison with their conventional counterparts in three ways. The 4-1BBL expressed on CAR-Ts self-interacts with the 4-1BB on these cells resulting in their enhanced *in vivo* persistence. 4-1BBL expressed on CAR-Ts also interacts with 4-1BB on the surface of DCs, inducing DC-secretion of IL-6 and IL-12. Additionally, CAR-T-expressed 4-1BBL interacts with 4-1BB on the surface of endogenous T cells leading to MHC-mediated bystander tumoricidal reactions. 4-1BBL, 4-1BB ligand; CAR, chimeric antigen receptor; DC, dendritic cell; IL-18R, IL-18 receptor; MHC-I, major histocompatibility complex class I; NK, natural killer cells; TCR, T-cell receptor; TME, tumor microenvironment.

IL-36 isoforms are vastly expressed by the skin, lung, and gastrointestinal tract epithelial cells in response to damage and they mediate the expression and secretion of more IL-36 through a positive feedback by acting on the IL-36 receptor (IL-36R) on epithelial cells and other immune system cells (such as DCs, monocytes, neutrophils, and T lymphocytes) which triggers inflammatory responses (158–160). Moreover, researchers have demonstrated that IL-36 presence mediates the production and secretion of T lymphocyte-related inflammatory cytokines in a direct manner (158–160). These findings gave rise to the idea that genetically engineering CAR-Ts to deliver IL-36 in a site specific fashion can improve their proliferation and tumoricidal activity alongside triggering bystander antitumor responses (**Figure 5A**) (159, 160). Recently, Li et al. demonstrated that CAR-T-mediated secretion of IL-36γ can also contribute to the mounting of collateral antitumor effects through the activation of APCs and T cells nearly aborting the outgrowth of tumor cells with the loss of CAR-T target antigen (161). Furthermore, the autocrine action mode of IL-36γ has been known to enhance the functionality of these CAR-Ts in terms of proliferation, persistence, and tumor elimination capability in comparison with those of conventional CAR-Ts (161).

### 5.7 IL-18-Secreting CAR-Ts

IL-18 is a cytokine from the IL-1 cytokine family that is expressed and secreted by macrophages (162). IL-18 induces IFN-γ expression and secretion and demonstrates pleiotropic activity on various cells of the immune system (162). These characteristics of IL-18 have made it a great candidate for improving the tumoricidal activity of genetically manipulated T cell therapies while creating efficient bystander antitumor effects. In 2017, Hu and colleagues generated IL-18-secreting CAR-Ts and demonstrated that these cells were capable of mediating enhanced tumoricidal activity against CD19-expressing xenograft mouse models (163). In the same year, Chmielewski et al. reported that their IL-18-secreting CAR-Ts suppressed the tumor outgrowth in syngeneic pancreatic cancer and xenogeneic lung cancer preclinical mouse models in an enhanced manner in comparison with conventional CAR-Ts without engineered cytokine secretion (164). It is worth mentioning that these researchers indicated that the tumors in the mentioned preclinical models were refractory to conventional CAR-T therapy (164). Ultimately, these researchers concluded that IL-18-secreting CAR-Ts can be utilized for rendering large solid tumors susceptible to bystander immune system-facilitated antitumor responses based on the finding that IL-18-secreting CAR-T treatment in preclinical models was associated with an increased number of M1 macrophages and NKG2D^+^ NK cells and reduced number of M2 macrophages, Tregs, and CD103^+^ DCs (164). According to a study by Avanzi et al., IL-18-secreting CAR-Ts can trigger endogenous antitumor responses alongside being capable of TME modulation and exhibiting pronounced expansion and persistence (**Figure 5B**) (162). In detail, they demonstrated that IL-18-secreting CAR-Ts can meaningfully prolong long-term survival in preclinical mouse models of hematologic and solid tumor neoplasms (162). Additionally, Huang and co-workers investigated the impacts of exogenous IL-18 on CAR-T tumoricidal activity and reported that IL-18 enhances the tumoricidal activity of HER2-redirected CAR-Ts in immunodeficient mice (165). These researchers also indicated that IL-18 demonstrates beneficial characteristics in improving ACT responses based on the finding that IL-18 enhanced the tumor-targeting capacity of OVA-specific T lymphocytes (165). Moreover, using IL-18 receptor (IL-18R)-knockout condition in immunocompetent mice and CAR-Ts, Huang et al. reported that IL-18R-independent pathways are responsible for antitumor-improving activities of IL-18 (165).

### 5.8 4-1BB Ligand (4-1BBL)-Expressing CAR-Ts

4-1BBL is a molecule with immune stimulation characteristics interacting with the 4-1BB receptor in the process of antigen presentation in CD4^+^ T lymphocytes and CD8^+^ T lymphocytes (166). During this process, 4-1BBL activates the downstream signaling cascades of *NF-kB*, *c-Jun*, and *p38* that results in costimulatory signals to T lymphocytes leading to pleiotropic immune system responses (166).

Systemic delivery of ligands that activate costimulatory receptors for the activation of only tumor-reactive T cells may not be feasible since all T cells possess the capability of expressing such costimulatory receptors (167, 168). Moreover, systemic administration of mAbs for this aim may not also be significantly beneficial due to the inability of such therapeutics for targeting these costimulatory receptors on a specific population of T cells (169, 170). Some studies have demonstrated that immunization with tumor cells expressing B7.1, 4-1BBL, or OX40L can mediate robust T cell responses in preclinical mouse models (171–175). Such findings demonstrated that employing costimulation for triggering and amplifying antitumor T cell responses is an important approach.

Engineered expression of CD80 or 4-1BBL by CAR-Ts can support their ability for the activation of DCs and bystander T cells (**Figure 5C**) (176, 177). In particular, Stephan et al. reported that primary human T lymphocytes that expressed CD80 and 4-1BBL showed reactivity towards malignant cells deficient in the expression of costimulatory ligands and triggered robust outgrowth suppression of large and systemic malignancies in preclinical immunodeficient mouse models (176). Also, these researchers demonstrated that CD80- and 4-1BBL-expressing T cells exhibited enhanced expansion, cytokine expression, survival, and persistence *in vitro* and *in vivo* in preclinical mouse models in comparison with T cells without CD80 and 4-1BBL expression (176).

In another study, Zhao et al. reported that 4-1BBL^+^ CAR-Ts (with CD28 as the costimulatory domain and CD3ζ as the activation domain) exhibited pronounced tumor eradication capacity, which was correlated with the induction of IRF7/IFNβ pathway in these cells (177). Moreover, these researchers reported that 4-1BBL^+^ CAR-Ts mediated tumor elimination by modulating the TME in two ways (177). First, these CAR-Ts assist the targeted delivery of 4-1BB costimulation leading to trans-costimulation (by presenting the 4-1BBL costimulatory ligand on their surface) (176, 177). Second, they also contribute to the targeted delivery of IFN-β that could improve tumor elimination through various mechanisms including improving DCs’ cross-priming functionality, suppressing Treg activation and expansion, and disturbing the microvasculature of the tumor (177). Moreover, Yang et al. studied the delivery IFN-β in a targeted manner using IFN-β-antibody constructs, and reported that this method boosted the cross-presentation of tumor antigens by CD8α DCs, resulted in the activation of CD8^+^ T lymphocytes, and mediated tumor outgrowth suppression (178). Such data further support that 4-1BBL^+^ CAR-Ts are capable of mediating endogenous bystander antitumor reactions, expanding the territory of target antigens beyond the CAR-T-targeted antigen, and inducing antitumor immune reactions that surpass CAR-T cytotoxicity in terms of duration (178).

Recently, Park et al. reported the results of a Phase I open label first-in-human clinical trial (NCT03085173) of CD19-redirected 4-1BBL^+^ CAR-Ts in patients with non-Hodgkin lymphoma and chronic lymphocytic leukemia for investigating the safety profile and overall response (OR) of these “*armored*” CAR-Ts (179). In detail, these researchers reported no severe case of cytokine release syndrome (CRS), and severe neurotoxicity was only documented in 8% of the enrolled patients (179). It was also reported that the CR rate was 57% with 11 out of 12 patients still in CR at the time of the report (179). Such outcomes pave the way for further clinical evaluations.

## 6 Alternative CAR-Expressing Effector T Cells

As discussed throughout the article, in recent years, various strategies have been developed for improving the outcomes of CAR-T therapy in solid tumors or to tackle its limitations. Some studies have proposed the employment of alternative effector T cells for CAR expression. Such CAR-expressing effector T cells can be beneficial for the treatment of solid tumors since these cells possess exclusive characteristics over conventional CAR-Ts. In the following section, we will briefly discuss two of these alternative effector T cells.

Natural killer T (NKT) cells are a type of MHC-independent cells originating from T lineage (180). NKT cells harbor morphological and functional features of both T cells and NK cells (180). Invariant natural killer T (iNKT) cells are a particular type of NKT cells that express the invariant TCR of Vα24Vβ11 capable of recognizing CD1d-presented glycolipid antigens (181). These cells act as a link between the innate immune system and the adaptive immune system (181). iNKT cells exhibit strong tumoricidal reactions by attacking and eliminating CD1d^+^ tumor cells, immunosuppressive TAMs, and MDSCs (67). Similar to NKT cells, iNKT cells are not human leukocyte antigen (HLA)-restricted, rendering them incapable of mediating Graft versus Host Disease (GvHD) (180, 181). Such characteristics make iNKT cells a suitable platform for CAR expression and solid tumor therapy. Of note, studies have demonstrated that there is a direct correlation between the number and proportion of tumor-infiltrating iNKT cells and the patient’s overall survival rate (182). For instance, one study has reported that higher iNKT cell infiltration in colorectal carcinomas is related to a more favorable patient prognosis (183). Another study has reported that the number of circulating iNKT cells can be a prognostic factor predicting the outcome of patients with head and neck squamous cell carcinoma (184).

CAR-modified iNKT (CAR-iNKT) cells have been considered as effector cells for CAR expression since it has been shown that these cells demonstrate exclusive antitumor functionality against various types of tumor cells (for instance in patients with lung cancer, head and neck cancer, or advanced melanoma) (185, 186). CAR-iNKT cells express particular chemokine receptors CCR1, CCR2, CCR4, CCR5, CCR6, and CXCR3 enabling their easy migration to the TME (187). Also, CAR-iNKT cells mediate cytolytic activity against CD1d-expressing TAMs and MDSCs (67). In detail, TAMs and MDSCs stimulate tumor outgrowth and keep tumor cells safe from various immune reactions through developing an immunosuppressive environment (67). Additionally, studies have demonstrated that CAR-iNKT cells coexpressing IL-15 or other cytokines exhibit enhanced survival in a TME with hypoxia, acidic conditions, and nutrient insufficiency (188). Overall, we hypothesize that CAR-iNKT cell therapy alongside immune checkpoint inhibitor therapies or with cytokine- or chemokine-equipped oncolytic virus therapy may result in CAR-iNKT cells with additionally enhanced tumoricidal effects through improved survival and migration.

Gamma delta (γδ) T cells are a specific type of T cells that harbor particular TCRs on their surface. A great proportion of T cells express α (alpha) and β (beta) TCR chains (known as αβ T cells) (189, 190). In contrast, the TCR of γδ T cells is composed of a γ chain and a δ chain (189, 190). These special T cells are about 1-5% of circulating lymphocytes and are the predominant type of lymphocytes in various poorly accessed places including the skin, reproductive system, and the intestine (189, 190). This characteristic of γδ T cells has made them ideal platforms for CAR expression and CAR-T therapy of various types of neoplasms, especially solid tumors (189, 190). γδ T cells express chemokine receptors which respond to chemokines produced and secreted by malignant cells (191). This action mechanism facilitates the migration of these T lymphocytes towards poorly accessed tumor sites (191). Moreover, Vγ9Vδ2 T cells are an important subset of γδ T cells capable of recognizing phosphoantigens (192, 193). This ability of Vγ9Vδ2 T cells is advantageous in targeting various types of tumor cells since tumor cells are known to be accumulators of certain phosphoantigens (192, 193). In regards to GvHD induction, similar to iNKT cell, the activation of γδ T cells is also independent of MHC molecules; therefore, allogeneic γδ T cells can be considered safe in terms of GvHD mediation (194, 195).

As mentioned earlier, the lack of definitive or highly expressed tumor antigens in solid tumor CAR-T therapy results in poor CAR-T stimulation, activation, persistence, and antitumor responses. In such cases, γδ T cells can be beneficial since they can act as proficient antigen-presenting cells following activation resulting in bystander antitumor effects (196). Additionally, γδ T cells can be an ideal type of effector cell for CAR-T therapy since they can be expanded to large numbers during *ex vivo* culture (194, 195). In a nutshell, γδ T cells possess various advantageous features that can benefit solid tumor CAR-T therapy in many ways.

## 7 Conclusion

The use of cutting-edge genetic engineering techniques as well as other combinatorial strategies has changed the face of the back-then novel concept of CAR-Ts which started in the late 1980s. Early CAR-Ts harbored an antibody fragment fused to T-cell activating domains for the redirection of specialized T cells against tumor cells of interest. Newer generations of this concept proved efficient in the treatment of various hematologic malignancies, but in the case of solid tumor treatment, they faced serious obstacles. Even though the unsuccessful outcomes of CAR-T therapy in solid tumors came as discouraging news, they encouraged researchers to develop novel strategies for enhancing CAR-T tumor infiltration rate, inducing cooperative endogenous antitumor effects, induced expression of CAR target antigens in tumor cells, and generating CAR-T boosting vaccines. These developed strategies have demonstrated that it might be feasible to overcome the mentioned biological barriers and to increase the efficacy and safety of CAR-T therapy for the treatment of solid tumors. However, more profound experiments in human trials can further validate the functionality of these strategies and shorten the distance between their preclinical success and their clinical applicability. Furthermore, tearing apart the complex building blocks of the pathophysiology of solid tumors will also give us significant flexibility to use them against the tumors themselves for a more effective cancer immunotherapy. Strategies, such as what we discussed in this review, might serve as a giant leap towards success for CAR-T therapy of patients with solid tumors; however, after broader meticulous preclinical and clinical investigations.

## Author Contributions

PouSK: Conceptualization, Investigation, Writing - original draft, Writing - review and editing, Validation, Supervision. PooSK: Conceptualization, Investigation, Writing - original draft, Writing - review and editing, Validation, Supervision. MAN: Conceptualization, Writing - review and editing. FY: Conceptualization, Writing - review and editing. SMJM: Conceptualization, Writing - review and editing. FR: Writing - review and editing, Validation. All authors contributed to the article and approved the submitted version.

## Conflict of Interest

The authors declare that the research was conducted in the absence of any commercial or financial relationships that could be construed as a potential conflict of interest.

## Publisher’s Note

All claims expressed in this article are solely those of the authors and do not necessarily represent those of their affiliated organizations, or those of the publisher, the editors and the reviewers. Any product that may be evaluated in this article, or claim that may be made by its manufacturer, is not guaranteed or endorsed by the publisher.
